# Effect of acupuncture therapy combined with fluticasone propionate in the treatment of persistent allergic rhinitis: study protocol for a randomized controlled trial

**DOI:** 10.1186/s13063-022-06020-6

**Published:** 2022-01-31

**Authors:** Qi Fan, Yixuan Feng, Yan Hou, Feihu Wu, Wei Zhang, Wenbin Nie, Bin Li, Zhongyu Zhou, Wenbin Fu, Lei Shi, Zhongren Sun, Hong Zhao

**Affiliations:** 1 Institute of Acupuncture and Moxibustion, China Academy of Chinese Medical Sciences, Beijing, 100700 China; 2 Luohu District Hospital of Traditional Chinese Medicine, ShenZhen, 518004 China; 3 Peking University, Beijing, 100871 China; 4 The First Affiliated Hospital of Anhui University of Chinese Medicine, HeFei, 230031 China; 5 The First Affiliated Hospital of Hunan University of Traditional Chinese Medicine, Changsha, 410000 China; 6 Beijing Hospital of Traditional Chinese Medicine, Capital Medical University, Beijing, 100010 China; 7 HuBei Provincial Hospital of Traditional Chinese Medicine, Wuhan, 430061 China; 8 Guangdong Provincial Hospital of Chinese Medicine, Guangzhou, 510120 China; 9 First Teaching Hospital of Tianjin University of Traditional Chinese Medicine, Tianjin, 300381 China; 10 The Second Hospital of HeiLongjiang University of Chinese Medicine, Harbin, 150009 China

**Keywords:** Persistent allergic rhinitis, Acupuncture therapy, Randomized controlled trial, Study protocol

## Abstract

**Background:**

Allergic rhinitis (AR) is an immunoglobulin E (IgE)-mediated inflammatory response.

Persistent allergic rhinitis (PAR) is a subtype of AR, but the treatment of PAR is still a problem. Acupuncture is used as an alternative therapy for AR in clinical practice. The aim of this study is to evaluate the effectiveness of acupuncture therapy combined with fluticasone propionate nasal spray in comparison to fluticasone propionate nasal spray alone in the relief of symptoms for PAR.

**Methods:**

This study is a multicenter, single-blind, randomized controlled trial. A total of 260 eligible patients will be randomly assigned into the treatment group or the control group. The treatment group will receive the nasal fluticasone propionate combined with acupuncture, and the control group will receive fluticasone propionate nasal spray alone for 6 weeks. The primary outcome is the change in the Reflective Total Nasal Symptom Score (rTNSS) from baseline to the end of treatment, and the Total Non Nasal Symptom Score (TNNSS), reflective total ocular symptom score (rTOSS), Rhinitis Quality of Life Questionnaire (RQLQ), use of antiallergic drugs, and the Rhinitis Control Assessment Test (RCAT) are used as secondary outcomes. The participants will be followed up for another 24 weeks after treatment.

**Discussion:**

This clinical trial will be able to provide high level evidence on the acupuncture therapy combined with fluticasone propionate nasal spray in the treatment of PAR.

**Trial registration:**

ISRCTN Registry, ID: ISRCTN44040506. Registered on 22 July 2020.

## Administrative information

Note: the numbers in curly brackets in this protocol refer to SPIRIT checklist item numbers. The order of the

items has been modified to group similar items (see http://www.equator- network.org/reporti ng- guideli nes/spirit-2013-stateme nt-defi nin g-sta ndard-protocol-items-for-cli ni cal-trials/).

**Table:** 

Title {1}	Effect of acupuncture therapy combined with fluticasone propionate in the treatment of persistent allergic rhinitis: study protocol for a randomized controlled trial.
Trial registration {2a and 2b}.	ISRCTN Registry, ID: ISRCTN44040506.Registered on 22 July 2020.
Protocol version {3}	Versio n 1.0 of 04-2020
Funding {4}	2019 National Administration of Traditional Chinese Project of building evidence based practice capacity for TCM-Project BEBPC- TC (NO.2019XZZX-ZJ); The Fundamental Research Funds for the Central public welfare research institutes (ZZ13-024-9)
Author details {5a}	Qi Fan: Institute of Acupuncture and Moxibustion, China Academy of Chinese Medical Sciences, China; Luohu District Hospital of Traditional Chinese Medicine,China Yixuan Feng: Institute of Acupuncture and Moxibustion, China Academy of Chinese Medical Sciences,China Yan Hou: Peking University,China Feihu Wu: The First Affiliated Hospital of Anhui University of Chinese Medicine,China Wei Zhang: The First Affiliated Hospital of Hunan University of Traditional Chinese Medicine, China Wenbin Nie: Institute of Acupuncture and Moxibustion, China Academy of Chinese Medical Sciences,China Bin Li: Beijing Hospital of Traditional Chinese Medicine, Capital
	Medical University,China Zhongyu Zhou: HuBei Provincial Hospital of Traditional Chinese Medicine,China Wenbin Fu, Guangdong Provincial Hospital of Chinese Medicine,China Lei Shi: First Teaching Hospital of Tianjin University of Traditional Chinese Medicine,China Zhongren Sun: The Second Hospital of HeiLongjiang University of Chinese Medicine,China Hong Zhao: Luohu District Hospital of Traditional Chinese Medicine,China
Name and contact information for the trial sponsor {5b}	Hong Zhao, hongzhao2005@aliyun.com
Role of sponsor {5c}	HZ is the project leader. She plays the role of study design and writing of the report. The funders have no role in designing or conducting this clinical trial.

## Introduction

### Background and rationale {6a}

Allergic rhinitis (AR) is defined as a symptomatic disorder of the nose resulting from an immunoglobulin E (IgE)-mediated immunological reaction to allergen exposure [1]. This highly prevalent inflammatory respiratory disease affects approximately 10–25% of the global population [2]. A study reported that the prevalence of self-reported AR in 11 Chinese cities ranges from 10 to 20% [3]. Classic symptoms of AR include rhinorrhea, nasal obstruction, nasal itching, and sneezing [4]. Comorbidities vary often, include sinusitis and asthma, have a significant impact on a person's quality of life, and are associated with sleep disorders, emotional problems, and social functioning [5, 6].

AR is traditionally classified as intermittent AR and persistent AR (PAR) depending upon symptoms duration [2]. The classification of AR severity is as mild and moderate/severe [7]. According to the different classifications, different treatment methods are recommended in the guidelines. Intranasal corticosteroid (INCS) is recommended as the first-line treatment for moderate/severe PAR [8]. Although the onset of its clinical effect appears very fast, the peak effect cannot be reached for several weeks in PAR cases [9]. In addition, the incidence of side effects, including burning, stinging, blood-tinged secretions, and dryness, ranges from 4 to 28%. The aftertaste, throat rundown, and nose run out of INCS also influences the preference and adherence of AR patients [10]. The current pharmacologic treatments for patients with PAR could still not achieve the expected benefits [11]. Moreover, clinicians may offer combination therapy in patients with persistent symptoms [7, 12].

Acupuncture treatment for patients with AR is widely used around the world. It is recommended that clinicians may offer acupuncture for patients with AR who are interested in nonpharmacologic therapy in the guidelines [11]. Previous studies have proven that acupuncture shows benefits in improving the symptoms of patients with AR [13–15]. A multicenter study has shown that active acupuncture can alleviate the nasal symptoms of PAR more effectively than sham acupuncture or observation alone for the treatment of persistent allergic rhinitis [16]. Another study has shown that quality-of-life improvements were more pronounced in the acupuncture versus routine-care group [17].

Combination of acupuncture and medicine is more common in the treatment of AR in clinical practice [18]. A trial about seasonal allergic rhinitis (SAR) has demonstrated that acupuncture plus rescue medication led to greater improvements in symptom than sham acupuncture with rescue medication, but with no demonstrable between-group differences. There is still a lack of evidence on the combination of acupuncture and western medicine for PAR.

We have participated in an international multicenter randomized controlled trial (RCT) which confirmed that acupuncture is superior to placebo acupuncture and no active treatment [16]. Based on this study, we improved the intervention, taking warm-needling as the intervention. After that, some studies observed that warm needling is more effective in treating PAR than SAR, especially for moderate to severe PAR [19]. In addition, we also found that acupuncture plus INCS has a better post treatment effect than using INCS alone.

The hypothesis of this trial is that acupuncture therapy combined with fluticasone propionate nasal spray has greater effectiveness than fluticasone propionate nasal spray in reducing the nasal symptoms of PAR and that there is a better after-treatment effect.

### Objectives {7}

This study aims to evaluate the efficacy of acupuncture therapy combined with fluticasone propionate nasal spray in patients with moderate to severe PAR.

### Trial design {8}

**Fig. 1:**
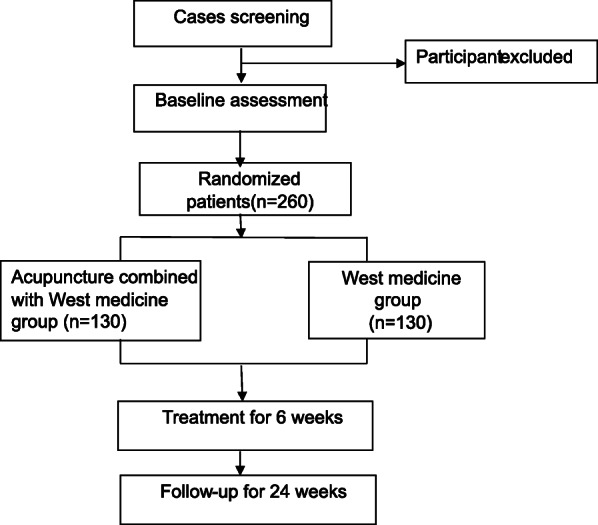
Trial flowchart

This study is a prospective multicenter, single-blind, two- arm, randomized controlled trial. A total of 260 eligible patients will be randomly assigned into the treatment group or control group with a 1:1 allocation ratio. The treatment group will receive nasal fluticasone propionate combined with acupuncture. The control group will receive fluticasone propionate nasal spray. Recruitment will begin on August 1, 2020, and will be completed on October 1, 2022. The trial flowchart is listed in Fig. 1.

## Methods: participants, interventions, and outcomes

### Study setting {9}

The study will be performed in nine hospitals or institution in China: (1) Institute of Acupuncture and Moxibustion, CACMS, (2) Beijing Hospital of Traditional Chinese Medicine, Capital Medical University, (3) The First Affiliated Hospital of Anhui University of Chinese Medicine, (4) Guangdong Provincial Hospital of Chinese Medicine, (5) The First Affiliated Hospital of Hunan University of Traditional Chinese Medicine, (6) HuBei Provincial Hospital of Traditional Chinese Medicine, (7) First Teaching Hospital of Tianjin University of Traditional Chinese Medicine, (8) The Second Hospital of HeiLongjiang University of Chinese Medicine, and (9) Luohu District Hospital of Traditional Chinese Medicine.

### Eligibility criteria {10}

The inclusion criteria:

- Age 18 to 65 years (either sex)
- Meet the diagnostic criteria for persistent allergic rhinitis [7]
- Meet the criteria for moderate to severe allergic rhinitis: the symptoms have a significant impact on quality of life, and the Reflective Total Nasal Symptom Score (rTNSS) score is greater than or equal to 6
- At least 2 consecutive years of history of persistent allergic rhinitis
- The participants provide written informed consent

The exclusion criteria:

- Participants who have suffered rhino sinusitis, respiratory diseases, acute paranasal sinusitis, or other systemic disease that may affect allergic rhinitis
- Participants who are allergic to smoke and dust produced by moxibustion
- Use of concomitant medications within the past 2 weeks that could interfere with study assessment, such as nasal/oral decongestants, nasal/oral antihistamines, mast cell membrane stabilizer, glucocorticoids or antileukotrienes
- Participants who have received specific immunotherapy or systemic hormone therapy within the past year
- Participants who have received the following treatments for allergic rhinitis in the past month: acupuncture, moxibustion, cupping, inhalation of traditional Chinese medicine in the nasal cavity, and other physical therapy treatments within the scope of traditional medicine, or who have received inhalation therapy and other medicine external therapy or other therapies
- Participants who have severe cardiovascular diseases or endocrine diseases

The practitioner of acupuncture treatment should meet the criteria as following:

- Qualified as a medical practitioner
- More than 2 years’ experience in acupuncture and moxibustion

### Who will take informed consent? {26a}

Research assistants are responsible for receiving patients who come for consultation. They are responsible for explaining the contents of informed consent, including research contents, patient rights and interests, etc. If there are some medical doubts, such as diagnosis and inclusion criteria, or the explanation is not clear, the researcher will take part in the informed consent process. After the patient has been assessed as eligible, he/she will sign the informed consent. The assistant will also be responsible for answering the participants’ questions and then transmitting the important information to the researchers during the study.

### Additional consent provisions for collection and use of participant data and biological specimens {26b}

There are no additional consent provisions foe collection and use of participant data and biological specimens.

### Interventions

#### Explanation for the choice of comparators {6b}

Fluticasone propionate is one of the effective topical anti-inflammatory corticosteroid. Large clinical studies involving PAR and seasonal allergic rhinitis confirmed the efficacy of this drug in reducing nasal symptoms. Comparing with oral antihistamines, it trend towards better efficacy. Fluticasone propionate administered once daily also offers a convenient and effective treatment. It was widely used in clinic. Then, it was chosen as the comparator [20].

All drugs required for the clinical trial was stored by researchers. The researchers will assign them randomly to the subjects and introduce the dose, time, and method of administration in detail.

#### Intervention description {11a}

Participants in the control group will be treated with fluticasone propionate nasal spray. Fluticasone propionate nasal spray (FLIXONASE®, GlaxoSmithKline, S. A.) will be used in a dose of 100 μg per daily (1 spray 50 μg per nostril once daily) in the morning. The doctor will guide the patient in the correct method of using fluticasone to avoid spraying towards the nasal septum.

Participants in the treatment group will be treated with fluticasone propionate nasal spray combined with acupuncture therapy. The acupuncture therapy will include 16 sessions of acupuncture and moxibustion in 6 weeks: three times a week in the first 4 weeks and twice a week in the 5th to 6th weeks. The control group will receive fluticasone propionate nasal spray alone once every day for 6 weeks. The two groups will be followed up for 24 weeks.

The selected acupoints are as follows: DaZhui (DU 14), YinTang (EX-HN 3), SiBai (ST 2), YingXiang (LI 20), ShangYingXiang (EX-HN 8), ChiZe (LU 5) bilateral, and Hegu (LI 4) (bilateral). All acupoints are localized according to WHO Standard Acupuncture Point Location. Warming needle will be applied at Dazhui point. After the needle is obliquely inserted at Dazhui (GV14), the lit moxa (diameter: 12 mm; length: 15 mm; Nanyang Lvying Moxibustion Product Co., Ltd., Nanyang, China) was placed 1.5–2 cm above the skin. When the first moxa stick has burnt out, the ashes will be removed and the second stick will be replaced following the same procedure. In total, two moxa sticks are used. Patients will feel a local thermal sensation, and the surrounding skin will become mildly red without any burning pain.

Each treatment session will be 30 min in duration. Acupuncture will be performed by licensed acupuncturists who have participated in the standardized operating procedure training. Sterile, disposable acupuncture needles (length: 25 to 40 mm; diameter: 0.25 mm; Hwato, Suzhou, China) will be used.

#### Criteria for discontinuing or modifying allocated interventions {11b}

The intervention is to be terminated in the case of severe adverse events, such as severe complications or hemorrhage or burns or unbearable acupuncture pain and unpermitted medication use.

#### Strategies to improve adherence to interventions {11c}

At the time of signing the informed consent, whole process of this study will be informed to the participants. The participants will be required to prepare enough time to participate in this study.

Researchers in each center will provide convenient treatment and evaluate time for patients to ensure that they can insist on treatment.

#### Relevant concomitant care permitted or prohibited during the trial {11d}

Patients will not be allowed to take any other additional complementary treatments during the trial period. If PAR symptoms were not adequately controlled during treatment, participants could be treated with H1 antihistamines or intranasal corticosteroids. All types of medicines, dosage, and usage will be recorded in the electronic case report form (eCRF).

#### Provisions for post-trial care {30}

If the participant suffers from adverse effects due to treatment, such as skin burns, we will provide follow-up suitable treatment until the patient recovers.

### Outcomes {12}

#### Primary outcome

The primary outcomes is the change in the Reflective Total Nasal Symptom Score (rTNSS) at week 6. The rTNSS assesses the sum of individual nasal symptom scores for nasal congestion, rhinorrhea, nasal itching, and sneezing in a reflective manner for the previous 12 h on a four-point scale (0 = absent symptoms, 1 = mild symptoms, 2 = moderate symptoms, and 3 = severe symptoms). The total score range is from 0 to 12.

The outcomes will be measured at baseline, weekly after treatment, at week 6 and follow-up (from the 10th week, 18th week, and 30th week from the start of treatment)

#### Secondary outcome

- Change in rTNSS from baseline at follow-up
- The TNNSS is determined in terms of the supplementary symptoms such as post nasal discharge, tearing, nasal or ocular itching, nasal or maxillary pain, and headache. It will be measured at baseline, weekly after treatment, week 6, and follow-up (from the 10th week, 18th week, and 30th week from the start of treatment)
- Reflective total ocular symptom score (rTOSS), including itchy or burning sensation, tearing and redness and is assessed on a 4-point scale (range, 0–3, with 0 absent; 1, mild, 2, moderate; and 3, severe). It will be measured at baseline, weekly after treatment, and at week 6 and follow-up (from the 10th week, 18th week, and 30th week from the start of treatment)
- Rhinitis Quality of Life Questionnaire (RQLQ), which has 28 questions in 7 domains (activity limitation, sleep problems, nose symptoms, eye symptoms, other symptoms, practical problems, and emotional function) ranked from 0 (no impairment) to 6 (severe impairment). RQLQ will be administered at baseline, after 6 weeks of the treatment, and at follow-up (from the 10th week, 18th week, and 30th week from the start of treatment)
- Reduction of rescue medication use will be measured using medication scores on a 3-point scale: 1-nasal/oral antihistamines, 2—nasal glucocorticoids, 3—oral glucocorticoids at baseline, every week during treatment, and follow-up (from the 10th week, 18th week, and 30th week from the start of treatment)
- Rhinitis control will be measured using the Rhinitis Control Assessment Test (RCAT). It is a 6-item questionnaire that surveys the patient’s impairments during the previous 7 days. The items are nasal congestion, sneezing, watery eyes, sleep problems, avoidance of certain activities, and self-perception of symptom control. Symptoms are assessed on a 5-point Likert scale from “1 = symptoms occur very frequently” to “5 = symptoms never occur.” RCAT will be measured at baseline, week 6 and follow-up

#### Participant timeline {13}

**Fig 2:**
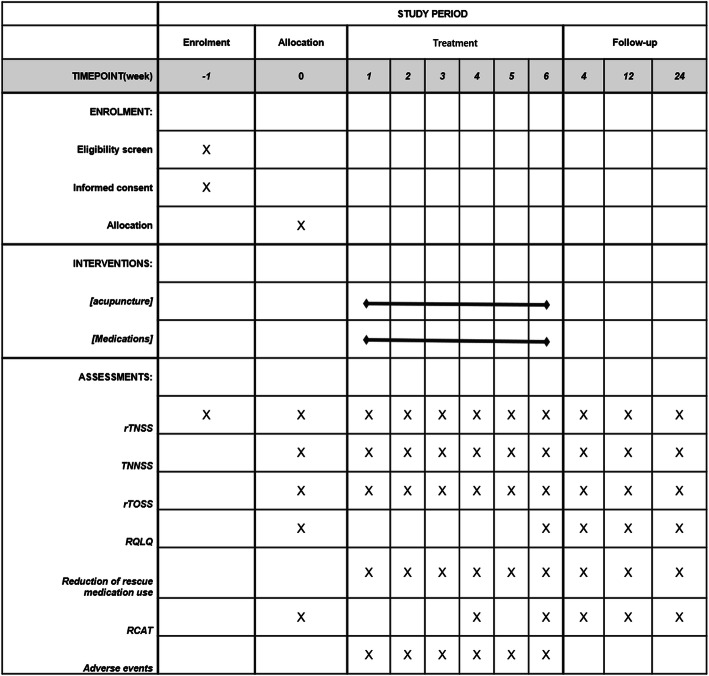
Study period

The schedule of enrolment, interventions, and assessments is in Fig. 2 (study period).

The duration of the study for each participant will be 31 weeks: 1 week before randomization as baseline and 6-week treatment and follow-up at the 10th, 18th, and 30th weeks.

#### Sample size {14}

The sample size was performed by statistical experts with PASS 15 (Kesville, UT, USA). The change in rTNSS at the end of treatment from baseline was used as the indicator for the efficacy evaluation in the sample size calculation. The result form a previous study showed that the mean change from baseline on rTNSS of fluticasone propionate in the treatment of PAR was 3.96 ± 2.26 [21]. Our pilot research showed that the change in rTNSS of acupuncture therapy combination with fluticasone was 5.33 ± 3.96. The superiority test was performed with superiority margin of 0.1, alpha of 0.025, statistical power of 0.8, and allocation ratio of 1:1. Therefore, considering the possibility of 20% drop-out rate, 260 participants will be recruited.

#### Recruitment {15}

All participants will be recruited from the nine hospitals or institution. Posters will be displayed on bulletin boards and hospital social media, and the interested subjects will contact the researchers. Information about the study, procedures, treatments, and possible risks will be carefully explained before enrollment. Patients who meet the inclusion criteria will be invited to participate in the study and will sign informed consent agreements. Basic information will also be collected by research assistants.

### Assignment of interventions: allocation

#### Sequence generation {16a}

According to the total sample size and allocation proportion, the Central Randomization System generates the allocation sequence by dynamic blocking randomization.

#### Concealment mechanism {16b}

The method of randomization is central competitive randomization. The Central Randomization System is provided by the Beijing Linkermed Tech Company which is an independent data management company. The researchers in different center will log into the random system to get random number and allocation group information.

#### Implementation {16c}

The Central Randomization System will generate the allocation sequence. Assistants in every center will enroll participants and will assign participants to interventions according to the allocation group information.

### Assignment of interventions: blinding

#### Who will be blinded {17a}

The method of randomization is central competitive randomization outcome assessors and statisticians will be blinded throughout the entire trial.

#### Procedure for unblinding if needed {17b}

The Central Randomization System is provided by the Beijing Linkermed Tech Company which is an independent data management company. The researchers in different centers will log into the random system to get random number and allocation group information.

### Data collection and management

#### Plans for assessment and collection of outcomes {18a}

The eCRF of this clinical trial is managed by a third-party corporation (Beijing Linkermed Tech Co, Ltd). Each sub-center has several dedicated clinical research coordinators (CRC), a clinical research associate (CRA), and a principal investigator (PI). Each user occupies a unique account in the system and grants different permissions to different researchers. All data will be collected by a CRC using the eCRF and input into the database, and the CRA will verify the data. The PI will conduct the quality control. No data will be permitted to be changed.

#### Plans to promote participant retention and complete follow-up {18b}

Sufficient follow-up information was explained by the researchers prior to the study. During the follow-up period, the research assistants will contact patients for collecting data of follow-up.

#### Data management {19}

The independent data monitoring committee (DMC) is made up of members from Peking University and conducts regular assessments of data quality and safety. A committee of Institute of Acupuncture and Moxibustion of CACMS will conduct at least one on-site sub-center visit every 6 months during the process of the trial to evaluate the consistency of acupuncture therapy and the authenticity of the eCRF.

#### Confidentiality {27}

Subject names will be abbreviated. All original medical records and study data will be treated as strictly confidential .These data will be retained with the researchers in Beijing Linkermed Tech Co, Ltd, and will not be handed over to any other party under any circumstances.

### Plans for collection, laboratory evaluation, and storage of biological specimens for genetic or molecular analysis in this trial/future use {33}

Not applicable.

### Statistical methods

#### Statistical methods for primary and secondary outcomes {20a}

The data verified by the Clinical Research Associate will be analyzed by statisticians using the Statistics Software SAS 9.4, and *P* ≤ 0.05 will be considered significant. For primary and secondary outcome measures, statistical data that have a normal distribution will be expressed by the mean ± standard deviation (*M* ± *SD*). Multivariate regression analysis as appropriate will be performed to analyze the outcome variable between the two groups, while the analysis of paired-sample *t* test will be used for the outcome variable within the two groups.

Effectiveness analyses for the primary outcomes will be performed in the FAS and the PPS population observed cases with the last observation carry-forward (LOCF) imputation of missing data.

#### Interim analyses {21b}

There are no interim analyses planned.

#### Methods for additional analyses (e.g., subgroup analyses) {20b}

Same as the analysis of statistical methods for primary and secondary outcomes

#### Methods in analysis to handle protocol non-adherence and any statistical methods to handle missing data {20c}

The researcher will contact participants as much as possible to supplement missing data. Furthermore, the missing data will be assessed using an intention-to-treat analysis.

#### Plans to give access to the full protocol, participant level-data, and statistical code {31c}

The datasets will be available after the trial is completed upon reasonable request and approval by the author.

### Oversight and monitoring

#### Composition of the coordinating center and trial steering committee {5d}

Principal investigator (PI) takes supervision of the trial. Clinical research coordinators (CRC) and clinical research associate (CRA) are responsible for the coordination and data management of each center. The study team, which consists of PI, CRC, and CRA, will meet and discuss the problems in the operation of the study every 3 months.

#### Composition of the data monitoring committee, its role and reporting structure {21a}

The independent data monitoring committee (DMC) is made up of members from Peking University and conducts regular assessments of data quality and safety.

#### Adverse event reporting and harms {22}

Adverse events will be monitored and recorded in the eCRF during the study. The possible side effects of acupuncture include burns, bleeding, hematoma, pain, and stuck needle. For all adverse events, necessary measures will immediately be taken by researchers for the safety of the participants.

#### Frequency and plans for auditing trial conduct {23}

The study team will meet every 3 months and will conduct at least one on-site subcenter visit every 6 months to check the quality of the data, including acupuncture operation, database operation, subject material, and informed consent.

#### Plans for communicating important protocol amendments to relevant parties (e.g., trial participants, ethical committees) {25}

Any substantial amendments after discussion by the expert group to the study protocol will be provided to ethics committees. In addition, online trial registries will be updated accordingly.

#### Dissemination plans {31a}

The trial results will be published in a peer-reviewed journal regardless of the outcome.

## Discussion

AR poses a global health problem and the incidence seems to have increased in recent years. The duration and severity of AR symptoms have a negative impact on a person’s quality of life and are also a significant societal burden [22–24]. Treatment of moderate to severe PAR remains a major problem in AR treatment. This type of patient needs long-term treatment, but the drug side effects are greater than for normal AR. There is considerable evidence that acupuncture therapy has a positive effect on improving allergic symptoms and quality of life [25, 26]. There is still a lack of evidence on the effectiveness of acupuncture plus first-line drugs in the treatment of PAR. Therefore, this proposed trial will investigate the effect of acupuncture therapy combined with fluticasone propionate nasal spray for treating moderate-severe PAR. According to WHO definition, acupuncture literally means to puncture with a needle. Its broad sense is to include traditional body needling, moxibustion, and laser acupuncture. Moxibustion [27] is a very important part of acupuncture therapy and is often used in combination with needling. In terms of mode of action, needling is a physical stimulus, while moxibustion is mainly a temperature stimulus. They play different roles in the prevention and treatment of disease. Moxibustion has the function of regulating the immune system. In the condition of immunosuppression, it could increase the number of T lymphocyte subsets in patients with cancer [28]. Acupuncture pretreatment can improve the exercise-induced immune imbalance caused by long-term excessive physical exercises [29]. On the other hand, it can interfere with the immune disorder of asthma and control the inflammatory reaction by decreasing CD8 +T and increasing the level of CD4 +T cells [30]. Therefore, on the basis of acupuncture, we add moxibustion treatment in order to obtain better therapeutic effect. This acupuncture plus moxibustion intervention was formulated by several acupuncture experts and has been used in clinical for more than 30 years. The pilot study also showed that it has good clinical effectiveness, especially with Western medicine, reducing the amount of western medicine, and has a better treatment after effect. Thus, we designed this clinical study. This clinical trial will be able to provide high level evidence on acupuncture therapy combined with fluticasone propionate nasal spray in the treatment of PAR. The results of this trial will provide better alternative choices for patients with PAR.

## Trial status

At the time of manuscript submission the study is currently in the recruitment phase. Recruiting started in October 1, 2020. Patient recruitment is estimated to be completed around October 31, 2021. Protocol version 1.0 of 10-04-2020.

## Abbreviations

AR: Allergic rhinitis
SAR: Seasonal allergic rhinitis
PAR: Persistent allergic rhinitis
TNSS: Total Nasal Symptom Score
TNNSS: Total Nonnasal Symptom Score
rTOSS: Reflective total ocular symptom score
RQLQ: Rhinoconjunctivitis Quality of Life Questionnaire
CACMS: China Academy of Chinese Medical Sciences
eCRF: Electronic case report form
DMC: Data monitoring committee
CRC: Clinical research coordinators
CRA: Clinical research associate
PI: Principal investigator
